# RNA-Seq Analysis Reveals an Essential Role of the Tyrosine Metabolic Pathway and Inflammation in Myopia-Induced Retinal Degeneration in Guinea Pigs

**DOI:** 10.3390/ijms222212598

**Published:** 2021-11-22

**Authors:** Ling Zeng, Xiaoning Li, Jian Liu, Hong Liu, Heping Xu, Zhikuan Yang

**Affiliations:** 1 Aier School of Ophthalmology, Central South University, Changsha 410000, China; zengling1128@163.com; 2 Aier Eye Hospital, Changsha 410000, China; lixiaoning@aierchina.com; 3 Aier School of Optometry and Vision Science, Hubei University of Science and Technology, Xian-ning 437100, China; 4 Aier Institute of Optometry and Vision Science, Changsha 410000, China; liujian2008@126.com (J.L.); 18670090456@163.com (H.L.); 5 The Wellcome-Wolfson Institute for Experimental Medicine, School of Medicine, Dentistry and Biomedical Sciences, Queen’s University Belfast, Belfast BT9 7BL, UK

**Keywords:** form deprivation, myopic retinopathy, animal model, RNA sequencing, complement cascade, ABC transporter, lipid biosynthesis, glycosaminoglycan biosynthesis

## Abstract

**Background:** Myopia is the second leading cause of visual impairment globally. Myopia can induce sight-threatening retinal degeneration and the underlying mechanism remains poorly defined. We generated a model of myopia-induced early-stage retinal degeneration in guinea pigs and investigated the mechanism of action. **Methods:** The form-deprivation-induced myopia (FDM) was induced in the right eyes of 2~3-week-old guinea pigs using a translucent balloon for 15 weeks. The left eye remained untreated and served as a self-control. Another group of untreated age-matched animals was used as naïve controls. The refractive error and ocular biometrics were measured at 3, 7, 9, 12 and 15 weeks post-FDM induction. Visual function was evaluated by electroretinography. Retinal neurons and synaptic structures were examined by confocal microscopy of immunolabelled retinal sections. The total RNAs were extracted from the retinas and processed for RNA sequencing analysis. **Results:** The FDM eyes presented a progressive axial length elongation and refractive error development. After 15 weeks of intervention, the average refractive power was −3.40 ± 1.85 D in the FDM eyes, +2.94 ± 0.59 D and +2.69 ± 0.56 D in the self-control and naïve control eyes, respectively. The a-wave amplitude was significantly lower in FDM eyes and these eyes had a significantly lower number of rods, secretagogin+ bipolar cells, and GABAergic amacrine cells in selected retinal areas. RNA-seq analysis showed that 288 genes were upregulated and 119 genes were downregulated in FDM retinas compared to naïve control retinas. In addition, 152 genes were upregulated and 12 were downregulated in FDM retinas compared to self-control retinas. The KEGG enrichment analysis showed that tyrosine metabolism, ABC transporters and inflammatory pathways were upregulated, whereas tight junction, lipid and glycosaminoglycan biosynthesis were downregulated in FDM eyes. **Conclusions:** The long-term (15-week) FDM in the guinea pig models induced an early-stage retinal degeneration. The dysregulation of the tyrosine metabolism and inflammatory pathways may contribute to the pathogenesis of myopia-induced retinal degeneration.

## 1. Introduction

Myopia (short-sightedness) is the leading cause of preventable visual impairment in children globally [1]. It is now widely accepted that there is an epidemic of myopia in school-age children in the developed countries of East and Southeast Asia [2,3]. The prevalence of myopia has increased steadily in the past few decades. A recent study in Taiwanese schoolchildren reported that from 1983 to 2017, the prevalence of myopia increased from 5.37% to 25.41% in 7-year-old schoolchildren and from 30.66% to 76.67% in 12-year-old schoolchildren [4]. The prevalence of high myopia has also increased over time, from 4.37% in 1983 to 15.36% in 2017 in 15-year-old schoolchildren [4]. The incidence of myopia in a Japanese population (>40 years old) increased from 37.7% in 2005 to 45.8% in 2017 [5]. A more recent study reported that ~70% of Japanese aged between 34–59 years old have myopia and the incidence of high myopia is ~10% [6]. In China, the incidence of myopia among individuals who were born after the 1960s reached a peak of 80% in the 1980s and a recent retrospective study reported the myopia prevalence had increased from 23.13% in 5 years old to 82.83% in 11-year-old schoolchildren [7]. The increased incidence of myopia is known to be related to changes in lifestyle in recent decades, such as reduced daily duration of outdoor activities and increased near-work activities, particularly on electronic devices [4]. The epidemic of myopia will likely get worse in the coming years, particularly with the ongoing COVID-19 pandemic, which has significantly negatively impacted myopia development and progression [8,9,10]. It is estimated that by 2050, approximately one-half of the world’s population will have myopia and up to one-fifth of the myopia population will be highly myopic [11].

Myopia is associated with a spectrum of ocular degenerative conditions that may affect the choroid, the Bruch membrane and the neuronal retina. The common degenerative changes include posterior staphyloma, lacquer cracks, optic disc abnormalities, choroidal atrophy, choroidal neovascularization and macular degeneration (myopic maculopathy). These degenerative changes can occur in any area of the retina and collectively, they are named “pathologic myopia” [12,13]. Older age, higher myopic spherical equivalent, longer axial length [13] are associated with a higher prevalence of myopic maculopathy [14] and posterior staphyloma [15]. In addition, retinal lattice degeneration has also been frequently observed in moderate myopic eyes and it has been postulated that this type of retinal degeneration may contribute to the high prevalence of retinal detachment among moderate myopia patients [16]. The pathogenesis of myopia-induced retinal degeneration remains poorly defined although choroidal ischemia and intraocular inflammation have been described as contributing factors of myopic macular neovascularization [13].

Although the mechanism of myopia development has been studied extensively in man and various animal models (e.g., chick, mouse, guinea pig, tree shrew, rabbit, etc.) [17,18,19], there are scarce reports on the mechanism of myopia-induced retinal degeneration. In this study, we induced progressive and moderate myopia using a long-term (15 weeks) form-deprivation protocol in guinea pigs and found that the treated eyes had impaired visual function and a reduced number of retinal neurons. Further, RNA sequencing analysis uncovered the dysregulation of tyrosine metabolism and impaired glycosaminoglycan and lipid biosynthesis and increased inflammation in the myopic retina.

## 2. Results

### 2.1. Refraction Error and Ocular Parameters in Form-Deprivation Induced Myopia

The baselines of axial length, vitreous chamber depth (VCD) (Table 1), refractive error and corneal curvature (Table 2) were equivalent among the three groups. The axial length increased with age in all groups during the course of the study with the highest increments observed in the FDM group (Table 1). Three weeks after form deprivation, the axial length in the FDM group was significantly longer than the other two groups and the difference remained during the course of the study (Table 1). In line with this, the VCD (Table 1) and refractive errors (Table 2) were significantly increased in the FDM group at three weeks and the increments progressed further until the end of the study. The axial length in the FDM group was significantly negatively correlated with the changes in refractive error (*p* < 0.05; r^2^ = 0.456) but positively correlated with the changes in VCD (*p* < 0.05; r^2^ = 0.451). After 15 weeks of intervention, the average refractive error of the FDM group was −3.40 ± 1.85 D compared to +2.94 ± 0.59 D and +2.69 ± 0.56 D in the self-control and naïve nontreated controls, respectively (Table 2), more than 6 D difference between FDM eyes and control eyes. The axial length, VCD, refractive error and corneal curvature in the self-controls and naïve nontreated controls were comparable (Table 1 and Table 2).

### 2.2. Electroretinogram Response in FDM and Control Eyes

**Figure 1:**
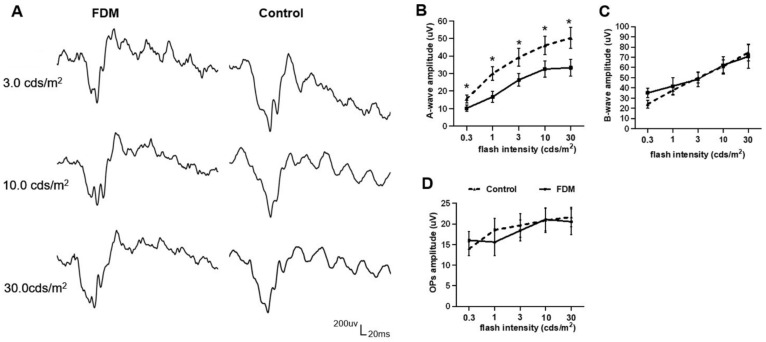
Electroretinography (ERG) in form-deprivation-induced myopia (FDM) and control eyes. (**A**) Representative scotopic ERG responses from FDM and naïve nontreated control eyes in 3.0 cds/m^2^, 10.0 cds/m^2^ and 30.0 cds/m^2^. (**B**–**D**) Statistical data of a-wave amplitude (**B**), b-wave amplitude (**C**) and OPs amplitude (**D**). Mean ± SD, *n* = 12/group. * *p* < 0.05. Multiple *t*-test.

Fifteen weeks after form deprivation, the a-wave amplitude in the FDM eyes was significantly reduced compared to the naïve nontreated control group (Figure 1A,B) in scotopic electroretinography (ERG). The b-wave amplitude and OPs amplitude did not differ between the two groups (Figure 1C,D). The a-wave implicit time of FDM eyes did not significantly differ from the control group. There was no difference in the a- and b-wave amplitudes and OPs between self-control and naïve control. The photopic a- and b-wave amplitude and implicit time between the three groups showed no significance (the data not shown).

### 2.3. Immunohistochemistry of Retinal Neurons in FDM and Control Eyes

#### 2.3.1. Photoreceptors

**Figure 2:**
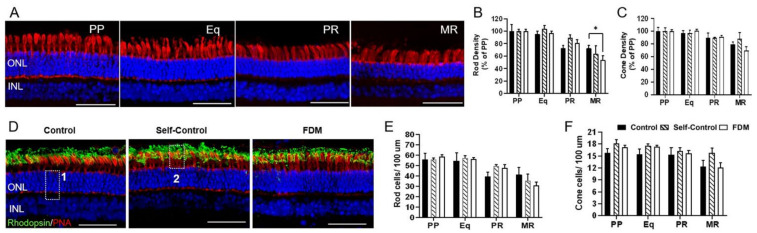
Photoreceptors in different groups of retinas. (**A**) Representative confocal images taken from the posterior pole (PP), equator (Eq), peripheral retina (PR) and marginal retina (MR) of peanut agglutinin (PNA)-labelled naïve control eye. Nuclei were stained with DAPI (blue). (**B**,**C**) The density of rod (**B**) and cone (**C**) expressed as % of PP in different retinal locations in naïve control, self-control and FDM eyes. (**D**) Representative confocal images of PNA (red) and rhodopsin (green) and DAPI labelled retinas from PP of naïve control, self-control and FDM eyes. Box 1 showing the counting of total number of photoreceptor nuclei; box 2 showing the counting of PNA^+^ cone cells. (**E**,**F**) The number of rod cells (**E**) and cone cells (**F**) in different retinal locations of naïve control, self-control and FDM eyes. ONL, outer nuclear layer; INL, inner nuclear layer. Scale Bar: 50 µm. Mean ± SD, *n* = 3~9/group. * *p* < 0.05. Two-way ANOVA followed by Fisher’s LSD test.

Cones were identified using the peanut agglutinin (PNA) staining and rhodopsin was used to stain rods. The density of rods and cones decreased from PP to MR in all groups (Figure 2A–C). The rod density in MR was 53% of the PP in FDM eyes, significantly lower than the MR rod density in the naïve control group (77% of the PP) (Figure 2B). The absolute number of rods and cones in the four locations (PP, Eq, PR, MR) did not significantly differ among the three groups (Figure 2D–F). We found no difference in the morphology and distribution of PNA and rhodopsin between different groups.

#### 2.3.2. Bipolar Cells and Synapses in Outer Plexiform Layer

**Figure 3:**
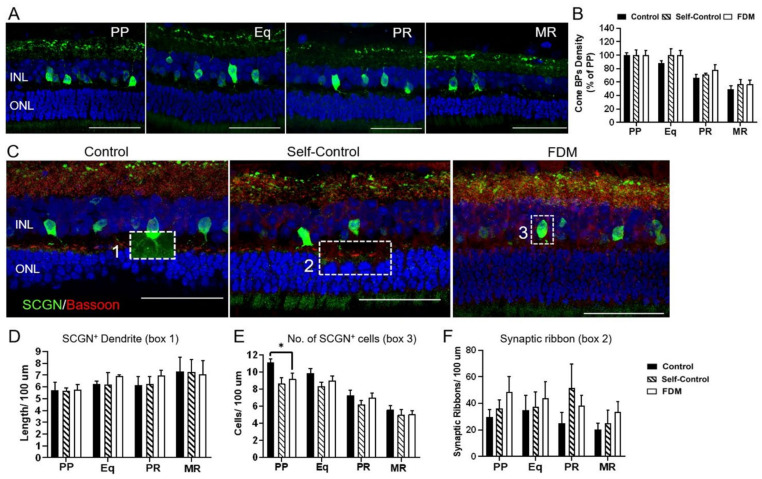
Cone-bipolar cells and synapses in outer plexiform layer. (**A**) Representative confocal images taken from the posterior pole (PP), equator (Eq), peripheral retina (PR) and marginal retina (MR) of naïve control eye staining for secretagogin^+^ (SCGN, green). Nuclei were stained with DAPI (blue). (**B**) SCGN^+^ cone-bipolar cells density expressed as % of PP in different retinal locations in naïve control, self-control and FDM eyes. (**C**) Representative confocal images of bassoon (red) and SCGN (green) labelled retinas from PP of naïve control, self-control and FDM eyes. Box 1 showing cone-bipolar dendrites; box 2 showing bassoon+ synaptic ribbon in the outer plexiform layer, box 3 showing SCGN^+^ cone-bipolar cell body. (D-F) Quantitative data of SCGN^+^ cone-bipolar dendrites (**D**), the number of SCGN^+^ cone-bipolar cells (**E**) and bassoon^+^ synaptic ribbons (**F**) in different retinal locations of naïve control, self-control and FDM eyes. INL, inner nuclear layer; ONL, outer nuclear layer. Scale Bar: 50 µm. Mean ± SD, *n* = 3~9/group. * *p* < 0.05. One-way ANOVA followed by Fisher’s LSD test.

Secretagogin (SCGN) and PKCα were used to identify cone- and rod-bipolar cells, respectively. The density of secretagogin^+^ cells decreased from PP to MR and there was no significant difference in the rate of reduction among the three groups (Figure 3A,B). The length of secretagogin^+^ dendrites (box 1 in Figure 3C) in different retinal locations in the three groups were comparable (Figure 3D). However, the number of secretagogin^+^ cells (box 3 in Figure 3C) in PP was significantly lower in FDM and self-control eyes compared to naïve control eyes (Figure 3E). There was no significant difference in the number and morphology of bassoon^+^ synaptic ribbons (box 2 in Figure 3C) in the three groups (Figure 3F).

**Figure 4:**
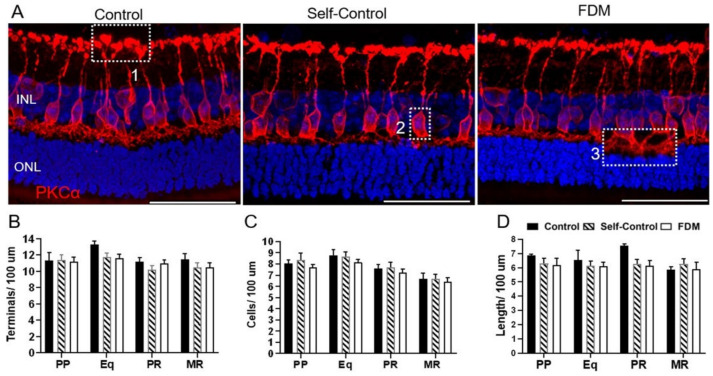
PKCα-bipolar cells and synapses in different groups of retinas. (**A**) Representative confocal images of PKCα labelled retinas from PP of naïve control, self-control and FDM eyes. Box 1 showing axon terminals; box 2 showing PKCα^+^ cell body, box 3 showing PKCα^+^ dendrites. (**B**–**D**) Quantitative data of PKCα^+^ terminals (**B**), the number of PKCα^+^ rod-bipolar cells and the length of PKCα^+^ rod-bipolar dendrites in different retinal locations of the three groups guinea pigs. INL, inner nuclear layer; ONL, outer nuclear layer. Scale Bar: 50 µm. Mean ± SD, *n* = 3~9/group.

The density of PKCα^+^ bipolar cells in the four retinal locations was similar in all groups (Figure 4). We did not detect any difference in the number of axon terminals (box 1 in Figure 4A), PKCα^+^ cells (box 2 in Figure 4A) and the length of dendrites (box 3 Figure 4A) between the different groups (Figure 4B–D).

#### 2.3.3. Horizontal Cells, Amacrine and Retinal Ganglion Cells

**Figure 5:**
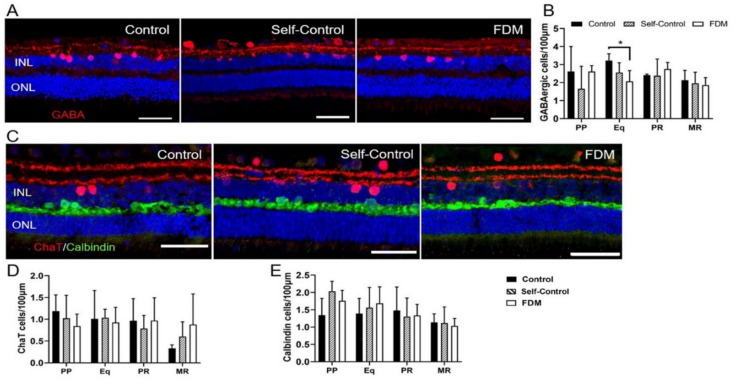
Horizontal cells and amacrine cells in different groups of retinas. (**A**) Representative confocal images of GABA labelled retinas from PP of naïve control, self-control and FDM eyes. Nuclei were stained with DAPI (blue). (**B**) Quantitative data showing the number of GABAergic amacrine cells (INL) in different retinal locations of the three groups guinea pigs. (**C**) Representative confocal images of CHAT (red) and Calbindin (green) labelled retinas from PP of naïve control, self-control and FDM eyes. Nuclei were stained with DAPI (blue). (**D**,**E**) Quantitative data showing the number of CHATergic amacrine cells (**D**) and calbindin^+^ horizontal cells in different retinal locations of the three groups of guinea pigs. INL, inner nuclear layer; ONL, outer nuclear layer. Scale Bar: 50 µm. Mean ± SD, *n* = 3~9/group. * *p* < 0.05. One-way ANOVA followed by Fisher’s LSD test.

The number of GABAergic amacrine cells from PP to MR did not change in all groups (Figure 5A,B). However, the number of GABAergic cells in Eq was significantly lower in FDM eyes compared to naïve controls (Figure 5B). We did not detect any significant changes in the number, distribution, and morphology of CHATergic amacrine cells among the three groups (Figure 5C,D). There was no significant difference in the number and distribution of calbindin^+^ horizontal cells (Figure 5C,E) in different groups.

**Figure 6:**
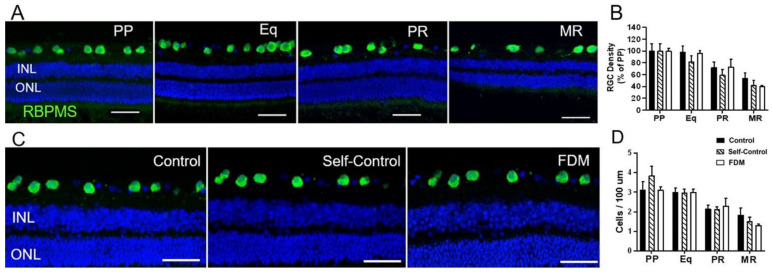
Retinal ganglion cells (RGC) in different groups of retinas. (**A**) Representative confocal images taken from the posterior pole (PP), equator (Eq), peripheral retina (PR) and marginal retina (MR) of naïve-control eye staining for RBPMS. Nuclei were stained with DAPI (blue). (**B**) The density of RGC expressed as % of PP in different retinal locations in naïve control, self-control and FDM eyes. PP of naïve control, self-control and FDM eyes. Nuclei were stained with DAPI (blue). (**C**) Representative confocal images taken from the PP of naïve control, self-control and FDM eyes showing RBPMS^+^ RGC. Nuclei were stained with DAPI (blue). (**D**) Quantitative data showing the number of RGCs in different retinal locations of the three groups of guinea pigs. Scale Bar: 50 µm. Mean ± SD, *n* = 3~9/group.

The density of RNA-binding protein with multiple splicing (RBPMS^+^) RGCs decreased from PP to MR in all groups (Figure 6A,B). The RGC density in MR was 40% of PP in FDM eyes, slightly but insignificantly lower than that in the naïve control eyes (53% of PP) (Figure 6B). Overall, there was no significant difference in the numbers of RBPMS^+^ RGCs in different groups (Figure 6C,D).

#### 2.3.4. Retinal Müller Glia and Microglial Cells

**Figure 7:**
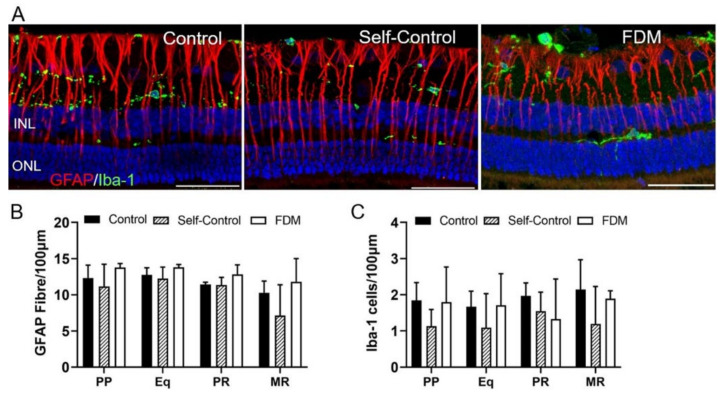
Retinal Müller glia and microglial cells. (**A**) Representative confocal images taken from the posterior pole (PP) of the retina stained for GFAP (red) and IBA-1 (green) in naïve control, self-control and FDM eyes. Blue—DAPI staining. (**B**,**C**) Quantitative data showing the number of GFAP^+^ Müller cells (**B**) and IBA-1^+^ microglial cells (**C**) in different retinal locations of the three groups. Scale Bar: 50 µm. Mean ± SD, *n* = 3~9/group.

GFAP^+^ Müller cells were found spinning from the nerve fiber layer to ONL (Figure 7A). There was no significant difference in the morphology and number of GFAP^+^ cells in the four retinal locations among the three groups (Figure 7A,B). IBA-1^+^ microglia were detected in the RGC, IPL, OPL, and occasionally in ONL (Figure 7A). There was no significant difference in the distribution, morphology and number of microglia in different groups (Figure 7A,C).

### 2.4. RNA-Sequencing Analysis of Molecular Changes in Myopic Retina

**Figure 8:**
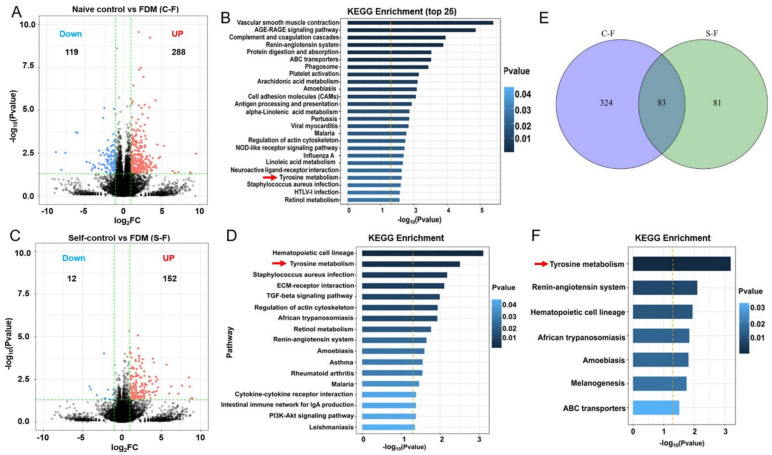
Transcriptional profiling of retinas from form-deprivation-induced myopic (FDM) guinea pigs. (**A**) Volcano plot showing highlights of differentially expressed genes (DEGs) from bulk RNA sequencing of retinas from naïve control and FDM animals. (**B**) The top 25 significantly enriched pathways in the FDM retina (compared to naïve control retina) from the KEGG analysis of the DEGs. (**C**) Volcano plot showing highlights of DEGs from bulk RNA sequencing of retinas from self-control and FDM animals. (**D**) The significantly enriched pathways in the FDM retina (compared to self-control retina) from the KEGG analysis of the DEGs. (**E**) Venn diagram showing the overlapping DEGs between “naïve control vs. FDM” and “self-control vs. FDM”. (**F**) The significantly enriched pathways in the FDM retina from the KEGG analysis of the 83 overlapping genes. Red arrows indicating the tyrosine metabolic pathway in three analysis.

The guinea pig reference genome (Ensembl_release100) were downloaded from ENSEMBL (http://asia.ensembl.org/Cavia_porcellus/Info/Index) on 1 April 2021. In our RNA-seq datasets, we detected 18,117 gene features in FDM retinas, 17,856 in self-control and 17,869 in naive control retinas. Considering that refractive error-induced retinal functional and cellular changes were mild in our study, we used the criteria of a fold change (FC) of at least 2 and *p* < 0.05 to determine the DEGs. Some 288 genes were upregulated and 119 genes were downregulated in FDM retinas compared to naive control retinas (Supplementary Table S1 DEG Ctr–vs. FDM, Figure 8A). The top 15 upregulated genes were related to signaling transduction (*WAS*, *SIX1*, *TIE1*, *RIPK3*), immune response (*WAS*, *FCN1*, *S100A9*, *S100A11*, *NLRC5*, *IOD1*), cell metabolism (*TECRL*, *SLC22A4*, *GZMK*, *TGM7*, *CHP2*) (Table 3). The top 15 downregulated genes were related to cytoskeleton organization (*CRYBA2*, *MYH8*, *CHAD)*, glucose/lipid metabolism (*SLC2A8*, *ALDH8A1*, *ACOT12*, *GRAMD2A*), and immune response (*BATF*, *DCST1*, *VMO1*, *Pol*, *IFITM5*) (Table 3).

We next performed KEGG enrichment analysis using the DEGs to identify functional pathways altered in the FDM retina. The significantly enriched pathways are related to inflammatory response, cellular metabolism and cardiovascular functions (Appendix A, Table A2). Out of the top 25 enriched pathways, 12 were related to inflammatory response and 7 were related to cellular metabolism (Figure 8B). When the up- and downregulated genes were analyzed separately, the AGE-RAGE signaling, complement cascades, NOD-like receptor signaling, IL-17 signaling and TNF signaling pathways were upregulated (Appendix A, Table A3), whereas antigen processing and cell adhesion pathways were downregulated (Appendix A, Table A4) in the inflammatory response category. In the cellular metabolism category, ABC transporters and tyrosine metabolism were upregulated (Appendix A, Table A3), whereas most of the lipid metabolic pathways including retinol metabolism were downregulated (Appendix A, Table A4).

When comparing the self-controls with FDM (S-F), 152 genes were upregulated and 12 genes were downregulated in FDM retina (Supplementary Table S2 DEG Self vs. FDM, Figure 8C). The significantly altered pathways identified in the KEGG enrichment analysis were also related to inflammation, cellular metabolism and cardiovascular function (Figure 8D). Interestingly, tyrosine metabolism was upregulated in the analysis of both C-F and S-F (red arrows in Figure 8B,D), whereas tight junction and glycosaminoglycan biosynthesis were downregulated in FDM retinas (Appendix A, Table A5).

When we compared the DEGs between C-F and S-F, 83 genes were shared by both groups (Figure 8E) and the majority of them changed in the same direction (Supplementary Table S3 Overlap DEG) indicating that these genes were differentially expressed in FDM eyes compared to self-control and naïve control. KEGG enrichment analysis of the 83 DEGs revealed seven significantly altered pathways, and the top one was the tyrosine metabolic pathway (red arrow, Figure 8F). In the tyrosine metabolic pathway, the expression of *TH* (tyrosine hydroxylase) was significantly reduced in FDM eyes compared to naïve controls (Log2(FC) = −0.35, *p* = 0.008), whereas *TYR*, *TYRP1*, *DCT* were significantly upregulated (Supplementary Table S3 Overlap DEG).

**Figure 9:**
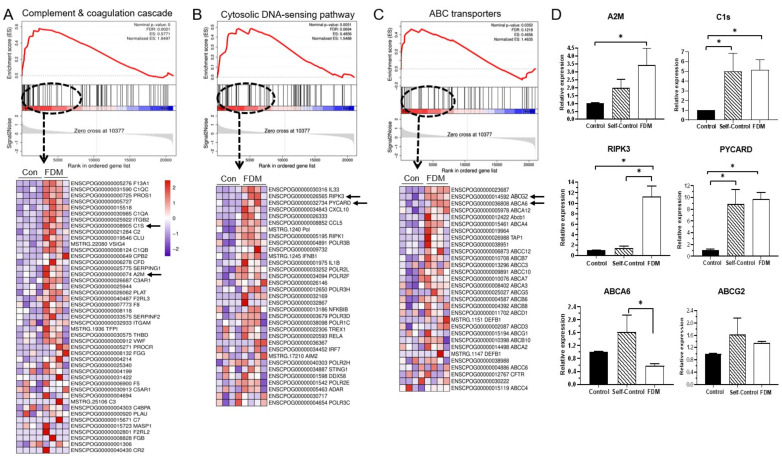
Gene set enrichment analysis (GSEA) of RNA-seq data from naïve control and FDM retinas. (**A**–**C**) GSEA enrichment plots and heat maps of leading edge genes of three gene clusters that are enriched in FDM retina were complement & coagulation cascade (**A**), cytosolic DNA-sensing pathway (**B**) and ABC transporters (**C**). Arrows indicating genes selected for qPCR verification. (**D**) Bar figures showing gene expression native control, self-control and FDM retinas. The value was expressed as gene fold change compared to naïve control. Mean ± SD, *n* = 6~8/group. * *p* < 0.05. One-way ANOVA followed by Fisher’s LSD test.

To further confirm the DEGs enriched pathways, we also conducted a Gene Set Enrichment analysis (GSEA) using the RNA-seq data from naïve control and FDM retinas. The complement and coagulation cascade, cytosolic DNA-sensing pathway and ABC transporters were top enriched pathways in the FDM retina with normalized enrichment scores of 1.95, 1.54 and 1.46, respectively (Figure 9A–C). We selected two genes from the leading genes of each pathway (arrows in the heat maps in Figure 9A–C) for qPCR verification. The expression of *A2M, C1S, RIPK3* and *PYCARD* was confirmed by qPCR, whereas *ABCG2* and *ABCA6* were not (Figure 9D).

**Figure A1:**
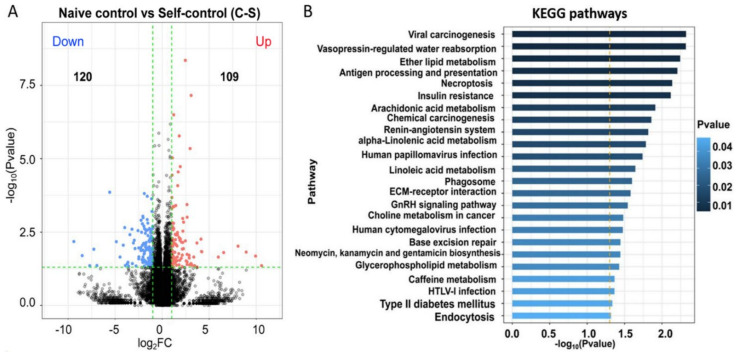
Transcriptional profiling of retinas from self-control eyes of FDM guinea pigs. (**A**) Volcano plot showing highlights of differentially expressed genes (DEGs) from bulk RNA sequencing of naïve control and self-control retinas. (**B**) The significantly enriched pathways in the self-control retina (compared to naïve control retina) from the KEGG analysis of the DEGs.

Considering that form deprivation in one eye may affect the visual function of the contralateral eye, we also conducted enrichment analysis in the DEGs between self-control and the naïve control retinas. We found that 109 genes were upregulated and 120 genes were downregulated in self-control retinas compared to naïve control retinas (Appendix B, Figure A1, Supplementary Table S4 DEG Ctr vs. Self). KEGG enrichment analysis identified 26 significantly altered pathways, including 13 up- and 13 down-regulated pathways (Appendix A, Table A6). The upregulated genes such as *RIPK3, PLA2G4E, PYCARD, PLD1, AQP4* and *AG1* are related to necroptosis, GnRH signalling pathway, choline metabolism and vasopressin-regulated water homeostasis (Appendix A, Table A6). Key down-regulated genes (i.e., *CREB5*, *SLC2A4*, *SREBF1*, *CYP1A1*, *CHARD* and *COMP*) are related to insulin resistance, response to virus infection, AMPK signalling and ECM receptor interaction (Appendix A, Table A6).

**Figure 10:**
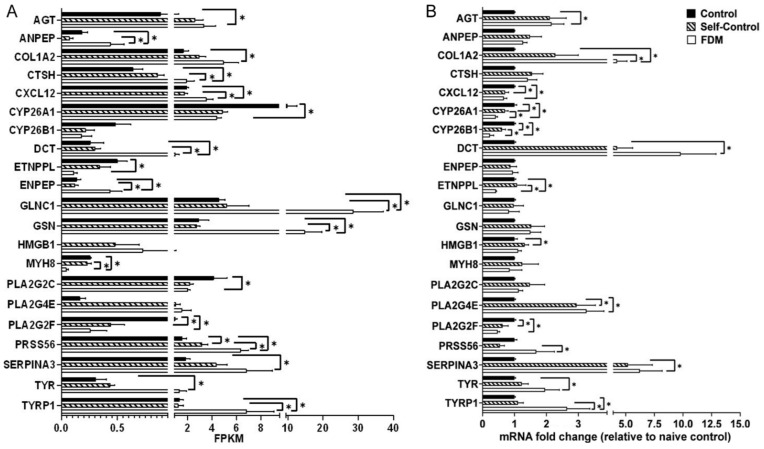
Verification of gene expression in naïve control, self-control and FDM eyes. (**A**) Histogram showing the FPKM (fragment per kilobase of transcript per million fragments) of representative DEGs among the three groups in RNA sequencing. (**B**) The relative gene expression measured by qPCR. Data presented as fold change of gene expression compared to native control. Mean ± SD, *n* = 4/group in (**A**), n = 6~8/group in (**B**). * *p* < 0.05. One-way ANOVA followed by Fisher’s LSD test.

Finally, we selected 21 DEGs (Figure 10A) that are involved in tyrosine metabolism, inflammation and lipid biosynthesis pathways and validated their expression using qPCR (Figure 10B). Ten genes (including three tyrosine metabolism-related genes, *TYR*, *TYRP1*, *DCT*) showed similar changes in RNA-seq and qPCR, 5 genes (*PLA2G2C, GSN, GLNC1, MYH8*, *ENPEP*, *ANPEP*, *CTSH*) were not confirmed by qPCR and one gene (*CXCL12*) showed opposite alteration in RNA-seq (up) and qPCR (down) (Figure 10).

## 3. Discussion

The pigmented guinea pigs are born with hyperopia, which decreases over time, but remain hyperopic in adulthood [20]. In our study, myopia was induced in 2–3-week-old guinea pigs and lasted for 15 weeks, equivalent to human age from 10 years old to ~30 years old (http://www.age-converter.com/guinea-pig-age-calculator.html, accessed on 17 November 2021). The 5.5- month-old guinea pigs were hyperopic for +2.69 ± 0.56 D, whereas the FDM animals had −3.40 ± 0.08 D myopia, a 6 D difference between control and FDM eyes. Our long-term (15 weeks) FDM protocol induced moderate to high myopia in the pigmented guinea pigs. Further electroretinography and immunofluorescent investigations showed that the FDM eyes had impaired visual function (reduced a-wave aptitudes) and mild retinal neuronal degeneration (lower number of rods, secretagogin bipolar cells and GABAergic amacrine cells in certain regions of the retina). Therefore, these animals could serve as a model of chronic myopia-induced early stage retinal degeneration.

Myopia-induced retinal degeneration is often sight threatening. Currently, there is no medication to prevent or treat the condition due to a lack of mechanistic insights. Retinal lattice degeneration, a localized thinning of the peripheral neuroretina, is most commonly found in patients with moderate myopia [16]. Reduced thickness of the retinal nerve fiber layer, particularly around the peripapillary, was present in the early stages of myopia [21]. The observations suggest that retinal neuronal degeneration may occur well before the clinical onset of myopic retinopathy. Recent studies using advanced imaging techniques such as optical coherence tomography angiography have reported an inverse association between eye size and retinal vasculature density in myopic eyes [22,23], suggesting a potential role of metabolic challenge in the pathogenesis of myopic retinopathy. Using RNA-seq technology, we found that many differentially expressed genes in the FDM retina were enriched in pathways related to immune responses such as the complement cascade (*C1s*, *C1QA*, *C1QC*, *ATGB2*, *A2M*), AGE-RAGE signaling pathway (*PLCG2*, *AGT*, *AGTR1*, *TGFB3*, *COL3A1*), cytosolic DNA-sensing (*IL-33*, *IRPK3*, *CXCL10* and *PYCARD*) and NOD-like receptor signaling pathway (*PSTPIP1*, *RIPK3*, *GBP1*, *PYCARD*, *Ifi204*). Inflammation is known to be involved in myopic retinopathy. Higher intraocular levels of CCL2 [24], IL6, MMP-2 and angiopoietin-1 [25,26] were detected in high myopic eyes. A recent proteomic analysis of aqueous humor from patients with pathological myopia reported increased innate immune response and complement activation [27]. The upregulation of the complement cascade-related genes has also been observed in the FDM in chicks [28]. A meta-analysis has suggested a role for the complement system in experimental myopia and hyperopia in chicks [29]. It is unclear whether the inflammatory responses are the consequence or the cause of myopic retinal degeneration.

Another important discovery of our study was the dysregulation of cellular metabolisms, including the tyrosine metabolic pathway, ABC transporters, lipid and glycosaminoglycan biosynthesize in the myopic retina. ABC transporters have important homeostatic roles in the retina, including blood–retinal barrier integrity [30] and the visual cycle [31]. Tyrosine is a nonessential amino acid that is critically involved in many biological functions. Tyrosine is essential for the generation of neurotransmitters such as dopamine, epinephrine and norepinephrine. Tyrosine also serves as a precursor of coenzyme Q10 (ubiquinone), a critical component of the mitochondrial electron transport chain that acts as an antioxidant. In addition, tyrosine is critically involved in the activation of various signaling transduction proteins through phosphorylation of the hydroxyl group of target proteins, the tyrosine kinases. Tyrosine kinases critically control the activation of signaling transduction of many growth factors such as VEGF, colony-stimulating factor-1 (CSF1), insulin and epidermal growth factor (EGFR) [32,33].

In mammals, tyrosine is synthesized from phenylalanine by phenylalanine hydroxylase (PHA). In our RNA-seq analysis, the *PHA* mRNA reads were zero in 11 retinas and 3 in one retina, indicating that the retina may not produce tyrosine in situ. Tyrosine hydroxylase (TH) is the primary enzyme responsible for catalyzing the conversion of tyrosine to L-3,4-dihydroxyphenylalanine (L-DOPA), the precursor of the neurotransmitters (e.g., dopamine, epinephrine) and melanin. TH is expressed in amacrine cells of human [34], zebrafish [35], chick, mouse, rat, guinea-pig and marmoset retinas [36]. TH immunoreactivity was also detected in a subset of inner plexiform cells in the cat retina [37]. In our study, the expression of *TH* was significantly reduced in myopic eyes compared to controls, suggesting that the tyrosine metabolism may be dysregulated in the myopic retina. The reduced tyrosine metabolism may be responsible, at least partially, for the myopia-induced retinal degeneration observed in this study.

We found that the gene expression of tyrosinase (*TYR*) and its stabilizers tyrosinase-related protein 1 (*TYRP1*) and tyrosinase-related protein 2 (*TYRP2*) were significantly upregulated in the myopic retina. A previous study has shown that in the absence of TH, the catecholamine neurotransmitters can be synthesized by tyrosinase [38]. However, tyrosinase is normally produced by melanocytes and retinal pigment epithelial (RPE) cells [39] but not neurons. Neurons may express tyrosinase (TYR) when TH is absent under disease conditions. The fact that these genes (*TYR*, *TYRP1* and *TYRP2*) were also detected in retinal samples from control nonmyopic eyes suggests the likely RPE contamination. Indeed, we detected *RPE65* mRNA in all of our retinal preparations. A previous study has shown that the uveal tyrosinase-dependent dopaminergic system is critically involved in myopia development and tyrosinase inhibition accelerated myopia development in pigmented guinea pigs [20]. Our results suggest that the tyrosinase pathway may play a role in myopia-induced retinal degeneration, for example through melanogenesis (see below).

Melanin has important homeostatic roles in the visual system due to its functions in light absorption, neutralization of free radicals [40] and regulation of inflammation [41]. A recent study has shown that melanin distribution in RPE cells is reduced in patients with early stages of myopic maculopathy without choroidal neovascularization or patchy chorioretinal atrophy and the reduction is associated with impaired retinal sensitivity [42]. This result suggests that melanin dysfunction in RPE cells contributes to the early stages of retinal degeneration in myopic conditions. During melanogenesis, TYR converts tyrosine to dopaquinone, which is then converted to dopachrome and eventually becomes eumelanin, a major type of melanin in RPE cells [43]. The TYRP2 (dopachrome tautomerase, DCT) is a melanogenic enzyme essential for the synthesis of eumelanin from dopachrome. The upregulation of TYR, TYRP1 and TYRP2 (DCT) in FDM eyes suggests enhanced melanogenesis, which was highlighted in our KEGG enrichment analysis. The oxidative stress and inflammatory responses resulting from myopia-induced retinal degeneration may initiate melanogenesis in RPE cells as feedback and a protective response to maintain homeostasis.

## 4. Materials and Methods

### 4.1. Animals

Guinea pigs (Cavia porcellus, pigmented) were purchased from Hunan Taiping Biotechnology Co., Ltd. (Yiyan, China), and maintained at the Department of Laboratory Animals of Central South University. The illuminance of the animal facility was approximately 300 lux on a 12 h day/12 h night cycle, and the room temperature was maintained at 24–26 °C with 60% humidity. All animals had free access to food and water. The study protocols were approved by the Animal Care and Ethics Committee of the Central South University (Ref: 2021SYDW0026), and all procedures were performed according to the Association for Research in Vision and Ophthalmology (ARVO) statement for the Use of Animals in Ophthalmic and Vision Research.

### 4.2. Form Deprivation-Induced Myopia

Two- to three-week-old guinea pigs were randomly assigned to two groups (*n* = 42/group, 21 males, 21 females): form deprivation and naïve nontreatment controls. In the form-deprivation group, the right eyes were occluded with nontoxic balloons as translucent masks for 15 weeks (named as “FDM”, form-deprivation-induced myopia), and the fellow eyes remained unoccluded and served as “self-controls”. The right eyes of untreated normal guinea pigs were used as “naïve controls”. The size of the mask was adjusted in time to ensure the tightness and daily activities of animals were not affected. Refractive error and ocular biometrics were measured on post-treatment weeks 3, 7, 9, 12 and 15.

### 4.3. Measurement of Refractive Error and Ocular Biometrics

The spherical equivalent refractive error, keratometry, axial length and vitreous chamber depth (VCD) were measured at baseline (i.e., before the start of treatment (0 week)), and at 3, 7, 9, 12 and 15 weeks after treatment using protocols modified from previous reports [44,45]. The spherical equivalent refractive error was measured under minimal light condition independently by two optometrists who were blind to the identity of the eyes. Briefly, 30 min after 3 drops of 0.5% tropicamide (Santen, Osaka, Japan), optometrists performed streak retinoscopy and trial lens in hand-held awake animals. The spherical equivalent was measured 3 times. The corneal curvature was measured at the center of the cornea using infrared photo-keratometry 3 times. The average of the three measurements was used as the value.

Axial length and vitreous chamber depth (VCD) were measured using an A-scan ultrasound (Axis Nano, Quantel Medical, Clermont-Ferrand, France). Topical anesthesia was administered with 0.5% proparacaine hydrochloride (Alcon (China) Ophthalmic Product Co. Ltd., Beijing, China) before ultrasonic measurements. The ultrasound frequency was set at 10 MHz. Ten readings were recorded for each measurement to calculate a mean result. Immediately after the measurements, the masks were re-placed in the FDM eyes.

### 4.4. Electroretinography

Electroretinography (ERG) was conducted at the end of the study, i.e., 15 weeks after treatment (±2 days allowed) at the same time of day (between 10 a.m.–2 p.m.). Both scotopic and photopic ERG responses were evaluated. Guinea pigs were dark adapted overnight, and all procedures were conducted under dim red light (<1 lux). The animals were anesthetized with an intraperitoneal injection of pentobarbital sodium (40 mg/kg) and pupils dilated using 0.5% tropicamide phenylephrine followed by topical anesthesia with 0.5% proparacaine hydrochloride. A heating table (38 °C) was used to maintain body temperature. The ERG was recorded using the Roland Electrophysiological system (Roland Consult, Wiesbaden, Germany) with ring-shaped corneal electrodes. For each animal, five light intensities (from 0.3 to 30 cds/m^2^) were applied. The following parameters were measured: a-wave amplitude, b-wave amplitude, a-wave implicit time, b-wave implicit time, oscillatory potentials (OPs) amplitude (the summed amplitude of wavelets 2–3), and OPs implicit time (the summed implicit time of wavelets 2–3).

The photopic ERG was measured using 4 light intensities (from 0.3 to 10 cds/m^2^) after 10 min light adaption at 25 cds/m^2^. ERG signals were averaged from 50 responses at each intensity level, with flash frequency 0.8 HZ. We compared the responses of the FDM eyes (right eyes) with the right eyes of the naïve controls, and the self-control eyes (left eyes) with the left eyes of naïve controls.

### 4.5. Immunofluorescence

At the end of the study, animals were sacrificed by CO_2_ inhalation and eyes were collected and processed for further investigations. For immunofluorescence, the eyes were fixed in 4% paraformaldehyde (Solarbio Life Sciences, Beijing, China) for 24 h, and then transferred to a 15% sucrose (Sinopharm Chemical Reagent, Shanghai, China) solution followed by 30% sucrose solution. The anterior segments of the eyes were removed and the eyecups were embedded in optimum cutting temperature (OCT) media (Sakura Finetek, Torrance, CA). Cryosections (14 mm thickness) were collected from the level of (or near to) optic nerve head to evaluate myopia-induced retinal neuronal degeneration. Retinal sections were blocked with 2% bovine serum albumin (BSA, Solarbio Life Sciences, Beijing, China) with 10% goat serum (BOSTER, Guangzhou, China) in 0.5% Triton X-100 in PBS for 1h at room temperature. The samples were then incubated with primary antibodies (diluted in 2% BSA; Table 1) overnight at 4 °C, followed by appropriate fluorophore-conjugated secondary antibodies (Appendix A, Table A1) for 2 h at room temperature. After thorough washes, samples were incubated with 4′,6-diamidino-2-phenylindole (DAPI, 1:10,000; Cat. D8200; Solarbio Life Sciences) for 5 min to stain cell nuclei. All sections were examined by confocal microscopy (LSM 880, ZEISS, Oberkochen, Germany).

### 4.6. Morphological Analysis of Retinal Cells

**Figure A2:**
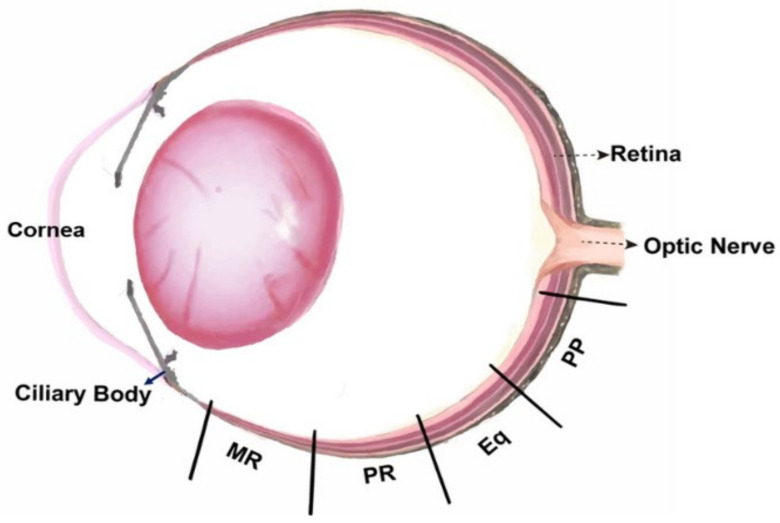
Diagram showing four locations from which confocal images were taken. PP, posterior pole; Eq: equator; PR: peripheral retina; MR: marginal retina.

Images from 4 locations of each retina (posterior pole (PP), equatorial (Eq), peripheral (PR) and marginal retina near the ciliary body (MR)) were used for retinal neuronal analysis (Appendix B, Figure A2). A minimum of two sections through the optic disc from 3~9 eyes/group were used in the retinal neuronal analysis. Confocal images were analyzed using ImageJ (Version: 1.4.3.67, National Institutes of Health, Bethesda, MD, USA). The measurements of cell number and synaptic structures were normalized to 100 µm retinal length. We carried out the following analysis: (1) rod and cone photoreceptor density (rods were calculated by subtracting the number of PNA^+^ cones from the total number of DAPI^+^ nuclei at the outer nuclear layer, ONL); (2) the density of bassoon^+^ synaptic ribbons; (3) the number of horizontal, rod-bipolar and cone-bipolar cells; (4) the length of rod- and cone-bipolar dendrites (measure from the apical border of the cell soma); (5) the number of GABAergic and CHATergic amacrine cells; (6) the number of RGCs; (7) Müller cell fiber density; (8) microglial cell density. All confocal images were independently analyzed by two researchers in a double-blind manner.

### 4.7. RNA Sequencing

RNA Extraction: Four eyes from each group were used for the RNA sequencing study. Retinas were dissected on ice within 2 min after enucleation of the eyeballs and slap frozen in liquid nitrogen and stored at −80 °C until used. Total RNA was extracted using a Trizol reagent kit (Invitrogen, Carlsbad, CA, USA) according to the manufacturer’s instructions. RNA quantity was measured using Qubit 2.0 and Nanodrop One (Thermo Fisher Scientific, MA, USA) at the same time. RNA integrity was determined by Agilent 2100 Bioanalyzer (Agilent Technologies, Palo Alto, CA, USA).

RNA sequencing: The RNA-seq was performed by Gene Denovo Biotechnology Co. (Guangzhou, China) using the Illumina NovaSeq6000 platform. Briefly, 1 µg mRNA was purified from total RNA using Oligo (dT)-attached magnetic beads (#E7530, New England Biolabs, Ipswich, MA). The mRNA was reverse transcripted into cDNA with random primers. Second-strand cDNA was synthesized by DNA polymerase I, RNase H, dNTP and buffer. The cDNA fragments were purified with QiaQuick PCR extraction kit (Qiagen, Venlo, Netherlands), end-repaired, poly(A) added, and ligated to Illumina sequencing adapters. The ligation products were size selected by agarose gel electrophoresis, PCR amplified and sequenced using the Illumina NovaSeq6000 platform.

Sequencing data preprocessing and analysis: The quality of the sequencing data was assessed with FASTQ [46] (Version 0.18.0). Clean reads were mapped to the ribosome RNA (rRNA) database to remove the rRNA sequences using Bowtie2 (Version 2.2.8). Reads with adapters, unknown nucleotides (N) >10%, containing A base and reads that half of the bases with Q score ≤ 20 were all filtered out. The remaining mRNA sequences were mapped to the genome (GCA_000151735.1) using HISAT2.2.4 [47]. Counts of each gene were extracted from the mapping files using StringTie [48] (v1.3.1). RNA differential expression between two different groups was analyzed using the DESeq2 software [49], which takes into account the length and number of genes. Data were analyzed to identify differentially expressed genes (DEGs) in a pairwise comparison between (1) self-control and FDM (S vs. F); (2) naive control and FDM (C vs. F); (3) naïve control and self-control (C vs. S). The empirical Bayes moderated t-statistic was used to determine the *p*-value of DEGs, which was corrected for multiple testing using the Benjamini–Hochberg method. Genes with the parameter of *p* < 0.05 and absolute fold change ≥2 were considered as DEGs, and those genes were used for Kyoto Encyclopedia of Genes and Genomes (KEGG) enrichment analysis and the pathways with *p* < 0.05 were considered to be significant. Additionally, considering the Gene Set Enrichment Analysis (GSEA) does not require an arbitrary cut-off for differential gene expression and it has a larger functional range [50], and the refractive error-related retinal gene expressional changes are often modest [51], we also performed GSEA in our datasets.

### 4.8. Quantitative Real-Time PCR (qRT-PCR)

Six to eight eyes from each group were used for the qRT-PCR validation. Total RNA was extracted using an RNA extraction kit (Omega Bio-Tek., Norcross, GA) according to the manufacturer’s instructions. A measure of 600 ng of RNA from each retina sample was converted to cDNA using a Reverse Transcription kit (Vazyme, Nanjing, China). The cDNA was diluted 1:10 for the subsequent qRT-PCR. qTR-PCR was conducted in 96-well plates with a lightcycle96@Real-Time PCR System (Roche, Germany) and each sample was triplicated. The primers were purchased from Tsingke Biotechnology (Beijing, China) and detailed in Appendix A, Table A1. Each reaction (10 µL volume) contained 500 nM primer, 2 µL cDNA, 5 µL SYBR 2X Master Mix (Vazyme, Nanjing, China) and 2 µL ddH_2_O and the amplification was conducted under cycling conditions: preincubation at 95 °C for 10 min, followed by 45 cycles of 95 °C for 10 s and 60 °C for 15 s (single acquire), and melting at 65 °C for 60 s. The relative expression of candidate genes was obtained using the comparative threshold cycle (2^−ΔΔCt^) method [52].

### 4.9. Other Data Analysis

Axial length, VCD, corneal curvature, refractive error, morphometric data and ERG responses (scotopic a-wave, b-wave and photopic a-wave, b-wave, OPs) were analyzed using one-way ANOVA, followed by pairwise comparisons using the Fisher’s least-significant difference (LSD) test. The Student *t*-test was used when comparing the difference between two groups. All data were expressed as mean ± SD. *p* < 0.05 was considered statically significant.

## 5. Conclusions

In this study, using a long-term (15 weeks) form-deprivation protocol, we generated a model of myopia-induced retinal degeneration in guinea pigs. The FDM eyes had reduced a-wave aptitudes in electroretinography and lower numbers of retinal neurons including photoreceptors, bipolar cells and amacrine cells. The model mirrors moderate myopia-induced early stages of retinal degeneration. Mechanistically, we found that the tyrosine metabolic pathway is dysregulated and inflammatory pathways, including the complement cascade, are activated in FAM eyes. The causal link between tyrosine metabolism, inflammation and myopic retinal degeneration warrants further investigation.

## Figures and Tables

**Table 1:** The change of axial length, vitreous chamber depth in different groups during the course of the study (*n* = 12/group).

	Axial Length (mm)						Vitreous Chamber Depth (mm)					
Weeks	0	3	7	9	12	15	0	3	7	9	12	15
Control	7.85 ± 0.10	8.14 ± 0.16	8.54 ± 0.13	8.63 ± 0.07	8.72 ± 0.07	8.74 ± 0.09	3.11 ± 0.05	3.13 ± 0.05	3.16 ± 0.05	3.18 ± 0.06	3.19 ± 0.06	3.20 ± 0.06
Self-Control	7.89 ± 0.21	8.12 ± 0.18	8.47 ± 0.27	8.54 ± 0.18	8.68 ± 0.18	8.68 ± 0.15	3.10 ± 0.11	3.12 ± 0.10	3.15 ± 0.13	3.13 ± 0.14	3.15 ± 0.10	3.13 ± 0.10
FDM	7.85 ± 0.15	8.35 ± 0.17 *	8.74 ± 0.24	8.86 ± 0.22 *	9.06 ± 0.23 *	9.12 ± 0.24 *	3.10 ± 0.06	3.27 ± 0.12 *	3.40 ± 0.20 *	3.45 ± 0.18 *	3.53 ± 0.22 *	3.51 ± 0.22 *

*, *p* < 0.05 compared to control group or self-control group. One-way ANOVA followed by Fisher’s least-significant difference (LSD) test.

**Table 2:** The change of refractive error and corneal curvature in different groups during the course of the study (*n* = 12/group).

	Refractive Error (D)					Corneal Curvature Radius (mm)		
Weeks	0	3	7	9	12	15	0	15
Control	3.80 ± 0.81	2.93 ± 1.18	3.02 ± 0.56	3.06 ± 1.10	2.40 ± 0.73	2.69 ± 0.56	3.43 ± 0.08	3.91 ± 0.09
Self-Control	3.08 ± 1.04	2.79 ± 1.24	2.49 ± 1.20	2.03 ± 0.73	2.18 ± 0.89	2.94 ± 0.59	3.45 ± 0.09	3.87 ± 0.12
FDM	2.94 ± 1.47	1.35 ± 1.87 *	0.38 ± 2.89 *	−0.66 ± 2.51 *	−2.28 ± 2.22 *	−3.40 ± 1.85 *	3.44 ± 0.08	3.92 ± 0.13

*, *p* < 0.05 compared control group or self-control group. One-way ANOVA followed by Fisher’s least-significant difference (LSD) test.

**Table 3:** Top 15 upregulated and downregulated genes in FDM retina compared to naïve control retina.

Top 15 Upregulated Genes				Top 15 Downregulated Genes			
Symbol	Log2(fc)	*P* Value	Description	Symbol	Log2(fc)	*P* Value	Description
*WAS*	7.229	0.041	WASP actin nucleation promoting factor	*BATF*	−8.077	0.030	BATF
*TECRL*	6.748	0.046	Trans-2,3-enoyl-CoA reductase like	*PHF23*	−7.570	0.003	PHD finger protein 23
*SIX1*	4.807	0.005	SIX homeobox 1	*SLC2A8*	−4.382	0.011	Solute carrier family 2, facilitated glucose transporter member 8-like
*FCN1*	4.605	0.006	Ficolin 1	*FAM186B*	−4.115	0.031	Family with sequence similarity 186 member B
*SLC22A4*	4.322	0.023	Solute carrier family 22 member 4	*ALDH8A1*	−3.907	0.015	Aldehyde dehydrogenase 8 family member A1
*TIE1*	4.209	0.016	Tyrosine kinase with immunoglobulin like and EGF like domains 1	*DCST1*	−3.719	0.022	DC-STAMP domain containing 1
*S100A9*	4.115	0.019	S100 calcium binding protein A9	*VMO1*	−3.700	0.019	Vitelline membrane outer layer 1 homolog
*S100A11*	3.886	0.021	S100 calcium binding protein A11]	*Pol*	−3.143	0.002	LORF2 protein, partial
*GZMK*	3.652	0.029	Granzyme K	*IFITM5*	−2.839	0.015	Interferon induced transmembrane protein 5
*NLRC5*	3.649	0.034	NLR family CARD domain containing 5	*SAMD13*	−2.807	0.028	Sterile alpha motif domain containing 13
*RIPK3*	3.629	0.014	Receptor interacting serine/threonine kinase 3	*CRYBA2*	−2.641	0.003	Crystallin beta A2
*TGM7*	3.502	0.000	Transglutaminase 7	*ACOT12*	−2.558	0.014	Acyl-CoA thioesterase 12
*SLC9C1*	3.492	0.013	Solute carrier family 9 member C1	*MYH8*	−2.466	0.000	Myosin heavy chain 8
*IDO1*	3.433	0.010	Indoleamine 2,3-dioxygenase 1	*GRAMD2A*	−2.459	0.004	GRAM domain containing 2A
*CHP2*	3.426	0.020	Calcineurin like EF-hand protein 2	*CHAD*	−2.415	0.017	Chondroadherin

## Data Availability

The data presented in this study are all contained within the main body and the Supplementary Materials of this article.

## Supplementary Materials

The following are available online at https://www.mdpi.com/article/10.3390/ijms222212598/s1.

## Appendix A

**Table A1:** Primary and secondary antibodies used in immunofluorescence study.

Antigen	Host	Product ID	Dilution	Source	Cell Location
**Primary Antibodies**					
Peanut agglutinin(PNA)-Biotin	-	019-19741	1:500	Sigma	Cone photoreceptors
Rhodopsin	Mouse	MAB5356	1:500	Millipore	Rod photoreceptors
Bassoon	Mouse	SAP7F407	1:50	Invitrogen	Synaptic ribbons
Secretagogin (SCGN)	Rabbit	HPA006641	1:100	Sigma	Cone bipolar cells
PKCα	Mouse	Sc-8393	1:300	Santa Cruz	Rod bipolar cells
Calbindin	Rabbit	TA342845	1:50	Origene	Horizontal cells
c-aminobutyric acid (GABA)	Rabbit	20094	1:200	Immunostar	GABAergic amacrine cells
Choline Acetyltransferase	Goat	AB144P	1:100	Millipore	CHATergic amacrine cells
RBPMS	Rabbit	Ab194213	1:500	Abcam	Retinal ganglion cells
GFAP	Mouse	MCA4734GA	1:300	Bio-Rad	Muller cells
IBA-1	Rabbit	019-19741	1:300	Bio-Rad	Microglial cells
**Secondary antibodies**					
Anti-mouse IgG (H+L), Alexa Fluor 594	Goat	A-11005	1:500	Invitrogen	
Anti-rabbit IgG (H+L), Alexa Fluor 488	Goat	A-11034	1:500	Invitrogen	
Anti-goat IgG (H+L), Alexa Fluor 647	Donkey	A-21447	1:500	Invitrogen	
Streptavidin-Daylight 594	-	ZF1010	1:500	Vector Laboratories	

**Table A2:** KEGG enriched pathways (All DEGs from naïve control vs. FDM).

Pathway	No of DEGs	*P* Value
**Immune Responses**		
AGE-RAGE signaling pathway	9	0.0001
Complement and coagulation cascades	7	0.001
Phagosome	11	0.004
Platelet activation	8	0.007
Amoebiasis	9	0.008
Cell adhesion molecules (CAMs)	9	0.009
Antigen processing and presentation	6	0.012
Pertussis	5	0.014
Viral myocarditis	8	0.014
Malaria	5	0.017
NOD-like receptor signaling pathway	8	0.019
Influenza A	8	0.020
Staphylococcus aureus infection	8	0.025
HTLV-I infection	10	0.027
Cytosolic DNA-sensing pathway	4	0.030
African trypanosomiasis	5	0.038
Hematopoietic cell lineage	7	0.041
Inflammatory mediator regulation of TRP channels	5	0.041
Systemic lupus erythematosus	8	0.042
Human cytomegalovirus infection	9	0.042
IL-17 signaling pathway	5	0.044
**Cellular metabolism**		
ABC transporters	5	0.003
Protein digestion and absorption	7	0.003
Arachidonic acid metabolism	5	0.008
alpha-linolenic acid metabolism	3	0.014
Regulation of actin cytoskeleton	9	0.018
Linoleic acid metabolism	3	0.022
Tyrosine metabolism	3	0.023
Thiamine metabolism	2	0.027
Retinol metabolism	4	0.027
**Cardiovascular function**		
Vascular smooth muscle contraction	11	0.000
Renin-angiotensin system	4	0.001
**Others**		
Neuroactive ligand–receptor interaction	12	0.023
Apelin signaling pathway	6	0.046

**Table A3:** KEGG enriched pathway (upregulated DEGs from naïve control vs. FDM).

Pathway	No. of DEGs	*P* Value
**Inflammatory Responses**		
AGE-RAGE signaling pathway	9	0.00002
Complement and coagulation cascades	7	0.0002
Amoebiasis	9	0.001
NOD-like receptor signaling pathway	8	0.004
Influenza A	8	0.004
Pertussis	5	0.005
Staphylococcus aureus infection	8	0.006
Malaria	5	0.006
Systemic lupus erythematosus	8	0.010
Hematopoietic cell lineage	7	0.011
Cytosolic DNA-sensing pathway	4	0.013
Phospholipase D signaling pathway	8	0.013
African trypanosomiasis	5	0.014
IL-17 signaling pathway	5	0.016
Rheumatoid arthritis	7	0.021
Platelet activation	6	0.021
Fc gamma R-mediated phagocytosis	6	0.025
TNF signaling pathway	5	0.035
Fc epsilon RI signaling pathway	5	0.040
Phagosome	7	0.046
**Cellular metabolism**		
ABC transporters	4	0.007
Tyrosine metabolism	3	0.012
Thyroid hormone signaling pathway	5	0.032
Choline metabolism in cancer	4	0.049
**Cardiovascular function**		
Renin-angiotensin system	4	0.000
Vascular smooth muscle contraction	7	0.003
Renin secretion	4	0.025
Dilated cardiomyopathy (DCM)	6	0.031
Regulation of actin cytoskeleton	7	0.033
Hypertrophic cardiomyopathy (HCM)	4	0.049
Apelin signaling pathway	5	0.050

**Table A4:** KEGG enriched pathway (down-regulated DEGs from Naive control vs. FDM).

Pathway	No of DEGs	*P* Value
**Metabolic Pathways**		
Retinol metabolism	3	0.002
Arachidonic acid metabolism	3	0.003
Type I diabetes mellitus	3	0.006
alpha-Linolenic acid metabolism	2	0.006
Linoleic acid metabolism	2	0.008
Glycerophospholipid metabolism	3	0.009
Protein digestion and absorption	3	0.010
Fat digestion and absorption	2	0.016
Tryptophan metabolism	2	0.018
Metabolic pathways	12	0.020
Ether lipid metabolism	2	0.020
Caffeine metabolism	1	0.026
Steroid hormone biosynthesis	2	0.041
**Inflammatory responses**		
Graft-versus-host disease	3	0.004
Antigen processing and presentation	3	0.009
Phagosome	4	0.017
Human papillomavirus infection	5	0.022
Allograft rejection	3	0.026
Autoimmune thyroid disease	3	0.033
Cell adhesion molecules (CAMs)	3	0.048
**Cardiovascular function**		
Vascular smooth muscle contraction	4	0.003
Viral myocarditis	3	0.037
**Senescence and proliferation**		
Cellular senescence	4	0.006
Chemical carcinogenesis	2	0.036

**Table A5:** KEGG enriched pathway (Self-control vs. FDM).

Enriched Pathways by Upregulated DEGs		
Pathway	No of DEGs	*P* Value
**Inflammatory responses**		
Hematopoietic cell lineage	6	0.003
Staphylococcus aureus infection	6	0.004
African trypanosomiasis	4	0.009
Amoebiasis	5	0.018
Rheumatoid arthritis	5	0.021
Asthma	4	0.022
PI3K-Akt signaling pathway	8	0.027
Malaria	3	0.028
Intestinal immune network for IgA production	4	0.032
Leishmaniasis	4	0.034
ECM-receptor interaction	3	0.036
Phagosome	5	0.040
Fc gamma R-mediated phagocytosis	4	0.040
TGF-beta signaling pathway	3	0.043
Wnt signaling pathway	4	0.044
**Cellular metabolism**		
Tyrosine metabolism	3	0.002
Retinol metabolism	3	0.014
**Cardiovascular function**		
Regulation of actin cytoskeleton	6	0.008
Renin-angiotensin system	2	0.019
Dilated cardiomyopathy (DCM)	4	0.046
**Enriched pathways by downregulated DEGs**		
**Pathway**	**No of DEGs**	***p* value**
Tight junction	2	0.009
Salmonella infection	2	0.010
Glycosaminoglycan biosynthesis	1	0.015
Pathogenic *Escherichia coli* infection	2	0.015

**Table A6:** KEGG enriched pathways by DEGs between naïve control vs. self-control.

Naïve Control vs. Self-Control: Upregulated Pathways		
Pathways	*P* Value	Genes
Necroptosis	0.003	ENSCPOG00000026565(RIPK3); ENSCPOG0000013435(PLA2G4E); ENSCPOG00000032734(PYCARD); ENSCPOG00000007538(-)
GnRH signaling pathway	0.004	ENSCPOG00000010721(PLD1); ENSCPOG00000015538(GNRH1); ENSCPOG00000013435(PLA2G4E)
Choline metabolism in cancer	0.005	ENSCPOG00000010721(PLD1); ENSCPOG00000004913(SLC22A4); ENSCPOG00000013435(PLA2G4E)
Base excision repair	0.010	ENSCPOG00000004970(-); ENSCPOG00000007538(-)
Vasopressin-regulated water reabsorption	0.013	ENSCPOG00000004446(AQP4); ENSCPOG00000000298(ARHGDIB)
Ether lipid metabolism	0.014	ENSCPOG00000010721(PLD1); ENSCPOG00000013435(PLA2G4E)
Neomycin, kanamycin and gentamicin biosynthesis	0.018	ENSCPOG00000010344(HK3)
Cytosolic DNA-sensing pathway	0.022	ENSCPOG00000026565(RIPK3); ENSCPOG00000032734(PYCARD)
Arachidonic acid metabolism	0.024	ENSCPOG00000034340(-); ENSCPOG00000013435(PLA2G4E)
Legionellosis	0.024	ENSCPOG00000032734(PYCARD); ENSCPOG00000033682(HSPA6)
Sulfur relay system	0.029	ENSCPOG00000039069(URM1)
Phospholipase D signaling pathway	0.044	ENSCPOG00000023212(AGT); ENSCPOG00000010721(PLD1); ENSCPOG00000013435(PLA2G4E)
Hypertrophic cardiomyopathy (HCM)	0.048	ENSCPOG00000011275(-); ENSCPOG00000023212(AGT)
**Naïve control vs. self-control: Downregulated pathways**		
**Pathways**	***p* value**	**Genes**
Insulin resistance	0.007	ENSCPOG00000013545(CREB5); ENSCPOG00000031830(SLC2A4); ENSCPOG00000002439(SREBF1)
Viral carcinogenesis	0.008	ENSCPOG00000001300(-); ENSCPOG00000013545(CREB5); ENSCPOG00000012203(-); MSTRG.24368(Patr-A)
Human papillomavirus infection	0.010	ENSCPOG00000001300(-); ENSCPOG00000013545(CREB5); ENSCPOG00000025029(COMP); ENSCPOG00000032331(CHAD); MSTRG.24368(Patr-A)
AMPK signaling pathway	0.011	ENSCPOG00000013545(CREB5); ENSCPOG00000031830(SLC2A4); ENSCPOG00000002439(SREBF1)
HTLV-I infection	0.015	ENSCPOG00000001300(-); ENSCPOG00000013545(CREB5); ENSCPOG00000012203(-); MSTRG.24368(Patr-A)
Retinol metabolism	0.021	ENSCPOG00000015596(CYP1A1); ENSCPOG00000021751(CYP26A1)
Caffeine metabolism	0.022	ENSCPOG00000019452(-)
Cellular senescence	0.023	ENSCPOG00000001300(-); ENSCPOG00000022941(-); MSTRG.24368(Patr-A)
Chemical carcinogenesis	0.026	ENSCPOG00000015596(CYP1A1); ENSCPOG00000019452(-)
Graft-versus-host disease	0.030	ENSCPOG00000001300(-); MSTRG.24368(Patr-A)
Type I diabetes mellitus	0.038	ENSCPOG00000001300(-); MSTRG.24368(Patr-A)
ECM-receptor interaction	0.041	ENSCPOG00000025029(COMP); ENSCPOG00000032331(CHAD)
Phagosome	0.050	ENSCPOG00000001300(-); ENSCPOG00000025029(COMP); MSTRG.24368(Patr-A)

## References

[1] Pascolini D., Mariotti S.P. (2012). Global estimates of visual impairment: 2010. Br. J. Ophthalmol..

[2] Dolgin E. (2015). The myopia boom. Nature.

[3] Morgan I.G., French A.N., Ashby R.S., Guo X., Ding X., He M., Rose K.A. (2018). The epidemics of myopia: Aetiology and prevention. Prog. Retin. Eye Res..

[4] Tsai T.-H., Liu Y.-L., Ma I.-H., Su C.-C., Lin C.-W., Lin L.L.-K., Hsiao C.K., Wang I.-J. (2021). Evolution of the Prevalence of Myopia among Taiwanese Schoolchildren: A Review of Survey Data from 1983 through 2017. Ophthalmology.

[5] Ueda E., Yasuda M., Fujiwara K., Hashimoto S., Ohno-Matsui K., Hata J., Ishibashi T., Ninomiya T., Sonoda K.-H. (2019). Trends in the Prevalence of Myopia and Myopic Maculopathy in a Japanese Population: The Hisayama Study. Investig. Opthalmol. Vis. Sci..

[6] Nakao S.-Y., Miyake M., Hosoda Y., Nakano E., Mori Y., Takahashi A., Ooto S., Tamura H., Tabara Y., Yamashiro K. (2021). Myopia Prevalence and Ocular Biometry Features in a General Japanese Population: The Nagahama Study. Ophthalmology.

[7] Long E., Wu X., Ding X., Yang Y., Wang X., Guo C., Zhang X., Chen K., Yu T., Wu D. (2021). Real-world big data demonstrates prevalence trends and developmental patterns of myopia in China: A retrospective, multicenter study. Ann. Transl. Med..

[8] Xu L., Ma Y., Yuan J., Zhang Y., Wang H., Zhang G., Tu C., Lu X., Li J., Xiong Y. (2021). COVID-19 Quarantine Reveals That Behavioral Changes Have an Effect on Myopia Progression. Ophthalmology.

[9] Wong C.W., Tsai A., Jonas J.B., Ohno-Matsui K., Chen J., Ang M., Ting D.S.W. (2021). Digital Screen Time During the COVID-19 Pandemic: Risk for a Further Myopia Boom?. Am. J. Ophthalmol..

[10] Wang J., Li Y., Musch D.C., Wei N., Qi X., Ding G., Li X., Li J., Song L., Zhang Y. (2021). Progression of Myopia in School-Aged Children after COVID-19 Home Confinement. JAMA Ophthalmol..

[11] Holden B.A., Fricke T.R., Wilson D.A., Jong M., Naidoo K.S., Sankaridurg P., Wong T.Y., Naduvilath T., Resnikoff S. (2016). Global Prevalence of Myopia and High Myopia and Temporal Trends from 2000 through 2050. Ophthalmology.

[12] Ohno-Matsui K., Lai T., Lai C.-C., Cheung C.M.G. (2016). Updates of pathologic myopia. Prog. Retin. Eye Res..

[13] Ohno-Matsui K., Wu P.-C., Yamashiro K., Vutipongsatorn K., Fang Y., Cheung C.M.G., Lai T.Y.Y., Ikuno Y., Cohen S.Y., Gaudric A. (2021). IMI Pathologic Myopia. Investig. Opthalmol. Vis. Sci..

[14] Wong Y.-L., Sabanayagam C., Ding Y., Wong C.-W., Yeo A.C.-H., Cheung Y.-B., Cheung G., Chia A., Ohno-Matsui K., Wong T.-Y. (2018). Prevalence, Risk Factors, and Impact of Myopic Macular Degeneration on Visual Impairment and Functioning Among Adults in Singapore. Investig. Opthalmol. Vis. Sci..

[15] Zheng F., Wong C., Sabanayagam C., Cheung Y., Matsumura S., Chua J., Man R.E.K., Ohno-Matsui K., Wong T., Cheng C. (2021). Prevalence, risk factors and impact of posterior staphyloma diagnosed from wide-field optical coherence tomography in Singapore adults with high myopia. Acta Ophthalmol..

[16] Celorio J., Pruett R.C. (1991). Prevalence of Lattice Degeneration and Its Relation to Axial Length in Severe Myopia. Am. J. Ophthalmol..

[17] Schaeffel F., Feldkaemper M. (2015). Animal models in myopia research. Clin. Exp. Optom..

[18] Ashby R. (2016). Animal Studies and the Mechanism of Myopia—Protection by Light?. Optom. Vis. Sci..

[19] Norton T.T., Siegwart J.T. (2013). Light levels, refractive development, and myopia—A speculative review. Exp. Eye Res..

[20] Jiang L., Zhang S., Chen R., Ma L., Wang X., Wen Y., Qu J., Zhou X. (2018). Effects of the Tyrosinase-Dependent Dopaminergic System on Refractive Error Development in Guinea Pigs. Investig. Opthalmol. Vis. Sci..

[21] Bin Lim H., Shin Y.-I., Lee M.W., Lee J.-U., Lee W.H., Kim J.-Y. (2020). Association of Myopia with Peripapillary Retinal Nerve Fiber Layer Thickness in Diabetic Patients Without Diabetic Retinopathy. Investig. Opthalmol. Vis. Sci..

[22] Khan M.H., Lam A.K.C., Armitage J.A., Hanna L., To C.-H., Gentle A. (2020). Impact of Axial Eye Size on Retinal Microvasculature Density in the Macular Region. J. Clin. Med..

[23] Hassan M., Sadiq M.A., Halim M.S., Afridi R., Soliman M.K., Sarwar S., Agarwal A., Do D.V., Nguyen Q.D., Sepah Y.J. (2017). Evaluation of macular and peripapillary vessel flow density in eyes with no known pathology using optical coherence tomography angiography. Int. J. Retin. Vitr..

[24] Zhu X., Zhang K., He W., Yang J., Sun X., Jiang C., Dai J., Lu Y. (2016). Proinflammatory status in the aqueous humor of high myopic cataract eyes. Exp. Eye Res..

[25] Zhang J.S., Da Wang J., Zhu G.Y., Li J., Xiong Y., Yusufu M., He H.L., Sun X.L., Ju T., Tao Y. (2020). The expression of cytokines in aqueous humor of high myopic patients with cataracts. Mol. Vis..

[26] Yuan J., Wu S., Wang Y., Pan S., Wang P., Cheng L. (2019). Inflammatory cytokines in highly myopic eyes. Sci. Rep..

[27] Xue M., Ke Y., Ren X., Zhou L., Liu J., Zhang X., Shao X., Li X. (2021). Proteomic analysis of aqueous humor in patients with pathologic myopia. J. Proteom..

[28] Giummarra L., Crewther S.G., Riddell N., Murphy M.J., Crewther D.P. (2018). Pathway analysis identifies altered mitochondrial metabolism, neurotransmission, structural pathways and complement cascade in retina/RPE/choroid in chick model of form-deprivation myopia. PeerJ.

[29] Riddell N., Crewther S.G. (2017). Novel evidence for complement system activation in chick myopia and hyperopia models: A meta-analysis of transcriptome datasets. Sci. Rep..

[30] Chapy H., Saubamea B., Tournier N., Bourasset F., Behar-Cohen F., Decleves X., Scherrmann J.-M., Cisternino S. (2016). Blood-brain and retinal barriers show dissimilar ABC transporter impacts and concealed effect of P-glycoprotein on a novel verapamil influx carrier. Br. J. Pharmacol..

[31] Zhong M., Molday L.L., Molday R.S. (2009). Role of the C Terminus of the Photoreceptor ABCA4 Transporter in Protein Folding, Function, and Retinal Degenerative Diseases. J. Biol. Chem..

[32] Ross A.H. (1991). Identification of tyrosine kinase Trk as a nerve growth factor receptor. Cell Regul..

[33] Iwashita S., Kobayashi M. (1992). Signal transduction system for growth factor receptors associated with tyrosine kinase activity: Epidermal growth factor receptor signalling and its regulation. Cell. Signal..

[34] Crooks J., Kolb H. (1992). Localization of GABA, glycine, glutamate and tyrosine hydroxylase in the human retina. J. Comp. Neurol..

[35] Jang Y.-J., Yu S.-H., Lee E.-S., Jeon C.-J. (2011). Two types of tyrosine hydroxylase-immunoreactive neurons in the zebrafish retina. Neurosci. Res..

[36] Ballesta J., Terenghi G., Thibault J., Polak J. (1984). Putative dopamine-containing cells in the retina of seven species demonstrated by tyrosine hydroxylase immunocytochemistry. Neuroscience.

[37] Oyster C.W., Takahashi E.S., Cilluffo M., Brecha N.C. (1985). Morphology and distribution of tyrosine hydroxylase-like immunoreactive neurons in the cat retina. Proc. Natl. Acad. Sci. USA.

[38] Rios M., Habecker B., Sasaoka T., Eisenhofer G., Tian H., Landis S., Chikaraishi D., Roffler-Tarlov S. (1999). Catecholamine Synthesis is Mediated by Tyrosinase in the Absence of Tyrosine Hydroxylase. J. Neurosci..

[39] Jimenez M., Kameyama K., Maloy W.L., Tomita Y., Hearing V.J. (1988). Mammalian tyrosinase: Biosynthesis, processing, and modulation by melanocyte-stimulating hormone. Proc. Natl. Acad. Sci. USA.

[40] Hu D.-N., Simon J.D., Sarna T. (2008). Role of Ocular Melanin in Ophthalmic Physiology and Pathology. Photochem. Photobiol..

[41] Mochizuki M., Sugita S., Kamoi K. (2013). Immunological homeostasis of the eye. Prog. Retin. Eye Res..

[42] Harimoto A., Obata R., Yamamoto M., Aoki N., Yamanari M., Sugiyama S., Kitano M., Fujita A., Minami T., Ueda K. (2021). Retinal pigment epithelium melanin distribution estimated by polarisation entropy and its association with retinal sensitivity in patients with high myopia. Br. J. Ophthalmol..

[43] Istrate M., Vlaicu B., Poenaru M., Hasbei-Popa M., Salavat M.C., Iliescu D.A. (2020). Photoprotection role of melanin in the human retinal pigment epithelium. Imaging techniques for retinal melanin. Rom. J. Ophthalmol..

[44] Howlett M.H., McFadden S.A. (2007). Emmetropization and schematic eye models in developing pigmented guinea pigs. Vis. Res..

[45] Howlett M.H., McFadden S.A. (2006). Form-deprivation myopia in the guinea pig (Cavia porcellus). Vis. Res..

[46] Chen S., Zhou Y., Chen Y., Gu J. (2018). Fastp: An ultra-fast all-in-one FASTQ preprocessor. Bioinformatics.

[47] Kim D., Langmead B., Salzberg S.L. (2015). HISAT: A fast spliced aligner with low memory requirements. Nat. Methods.

[48] Pertea M., Pertea G.M., Antonescu C.M., Chang T.-C., Mendell J.T., Salzberg S.L. (2015). StringTie enables improved reconstruction of a transcriptome from RNA-seq reads. Nat. Biotechnol..

[49] Love M.I., Huber W., Anders S. (2014). Moderated estimation of fold change and dispersion for RNA-seq data with DESeq2. Genome Biol..

[50] Subramanian A., Tamayo P., Mootha V.K., Mukherjee S., Ebert B.L., Gillette M.A., Paulovich A., Pomeroy S.L., Golub T.R., Lander E.S. (2005). Gene set enrichment analysis: A knowledge-based approach for interpreting genome-wide expression profiles. Proc. Natl. Acad. Sci. USA.

[51] Riddell N., Giummarra L., Hall N., Crewther S.G. (2016). Bidirectional Expression of Metabolic, Structural, and Immune Pathways in Early Myopia and Hyperopia. Front. Neurosci..

[52] Livak K.J., Schmittgen T.D. (2001). Analysis of relative gene expression data using real-time quantitative PCR and the 2(-Delta Delta C(T)) Method. Methods.

